# Leukocyte Telomere Length in HIV-Infected and HIV-Exposed Uninfected Children: Shorter Telomeres with Uncontrolled HIV Viremia

**DOI:** 10.1371/journal.pone.0039266

**Published:** 2012-07-16

**Authors:** Hélène C. F. Côté, Hugo Soudeyns, Anona Thorne, Ariane Alimenti, Valérie Lamarre, Evelyn J. Maan, Beheroze Sattha, Joel Singer, Normand Lapointe, Deborah M. Money, John Forbes, the CIHR Emerging Team in HIV therapy, aging (CARMA)

**Affiliations:** 1 Department of Pathology and Laboratory Medicine, Faculty of Medicine, University of British Columbia, Vancouver, Canada; 2 Women’s Health Research Institute, Vancouver, Canada; 3 Centre for Blood Research, Vancouver, Canada; 4 Unité d’immunopathologie virale, Centre de Recherche du CHU Sainte-Justine, Montreal, Canada; 5 Department of Microbiology & Immunology, Faculty of Medicine, Université de Montréal, Montreal, Canada; 6 Department of Pediatrics, Faculty of Medicine, Université de Montréal, Montreal, Canada; 7 CIHR Canadian HIV Trials Network, Vancouver, Canada; 8 Department of Pediatrics, Faculty of Medicine, University of British Columbia, Vancouver, Canada; 9 Children’s and Women’s Health Centre of BC, Vancouver, Canada; 10 Service des maladies infectieuses, CHU Sainte-Justine, Montreal, Canada; 11 Centre maternel et infantile sur le SIDA, CHU Sainte-Justine, Montreal, Canada; 12 BC Women’s Hospital, Vancouver, Canada; 13 Department of Obstetrics and Gynaecology, Faculty of Medicine, University of British Columbia, Vancouver, Canada; Mayo Clinic, United States of America

## Abstract

**Objectives:**

Nucleoside reverse transcriptase inhibitors (NRTIs) used in HIV antiretroviral therapy can inhibit human telomerase reverse transcriptase. We therefore investigated whether *in utero* or childhood exposure to NRTIs affects leukocyte telomere length (LTL), a marker of cellular aging.

**Methods:**

In this cross-sectional CARMA cohort study, we investigated factors associated with LTL in HIV -1-infected (HIV^+^) children (n = 94), HIV-1-exposed uninfected (HEU) children who were exposed to antiretroviral therapy (ART) perinatally (n = 177), and HIV-unexposed uninfected (HIV^−^) control children (n = 104) aged 0–19 years. Univariate followed by multivariate linear regression models were used to examine relationships of explanatory variables with LTL for: a) all subjects, b) HIV^+^/HEU children only, and c) HIV^+^ children only.

**Results:**

After adjusting for age and gender, there was no difference in LTL between the 3 groups, when considering children of all ages together. In multivariate models, older age and male gender were associated with shorter LTL. For the HIV^+^ group alone, having a detectable HIV viral load was also strongly associated with shorter LTL (p = 0.007).

**Conclusions:**

In this large study, group rates of LTL attrition were similar for HIV^+^, HEU and HIV^−^ children. No associations between children’s LTL and their perinatal ART exposure or HIV status were seen in linear regression models. However, the association between having a detectable HIV viral load and shorter LTL suggests that uncontrolled HIV viremia rather than duration of ART exposure may be associated with acceleration of blood telomere attrition.

## Introduction

Globally approximately 3 million children are born to HIV-infected women every year [1]. Treatment of the mother with antiretroviral therapy (ART) during pregnancy and of her child prophylactically after birth is recommended [2] and has greatly reduced mother-to-child transmission of HIV [3], [4]. Despite this, an estimated 2.5 million children live with HIV throughout the world in 2010 [5]. Nucleoside reverse transcriptase inhibitors (NRTIs) such as zidovudine (AZT) can readily cross the placenta [6], yet little is known about the possible long-term effects of *in utero* or early life exposure to NRTIs on HIV-exposed uninfected (HEU) children.

Telomeres cap and protect the end of chromosomes [7]. Telomerase is the enzyme complex responsible for replicating telomeres during cellular division and preventing telomere attrition [8]. Telomerase activity is present in stem cells, embryonic tissues and placenta, but is absent from most human somatic cells [9], with the exception of tissues that undergo rapid proliferation and can express telomerase transiently (*i.e.* germ line cells, epithelial cells, and hematopoietic cells) [10]. Despite this, peripheral blood cell telomeres do shorten as we age, acquire various infections and are exposed to stresses, which can eventually lead to immunosenescence in the elderly [11]. HIV infection itself can cause inflammation as well as chronic immune activation and proliferation of some blood cells, further shortening telomere length and potentially mimicking immunosenescence [12], [13]. In addition, telomere dynamics in HIV infection are complicated by the fact that telomerase comprises a reverse transcriptase that shares homology with HIV reverse transcriptase [14], [15]. NRTIs, the backbone of most HIV ART regimens, inhibit telomerase activity *in vitro* [16], [17], and can shorten telomeres in cultured cells [18], [19], [20], as well as in various model organisms [21], [22]. Indeed, AZT is used in adult acute T-cell leukemia chemotherapy [23], where it triggers cell senescence through telomere shortening [24].

The effect of HIV and NRTI exposure on telomere length in infants and children is largely unknown. There is accumulating evidence that HIV-infected individuals have a shorter life expectancy than their uninfected peers and that they are at higher risk for pathologies and complications typically associated with aging [13]. As shorter leukocyte telomere length (LTL) has been associated with increased risk of cardiovascular disease, cancer, and mortality [25], [26], [27], we investigated LTL and the factors associated with shorter LTL in HIV-1-infected children (HIV^+^), HEU children who were exposed to ART perinatally, and HIV-unexposed uninfected (HIV^−^) controls.

## Materials and Methods

### Study Design and Population

Subjects were enrolled in the prospective CARMA cohort at two sites: the Oak Tree Clinic at British Columbia (BC) Women’s Hospital and Health Centre in Vancouver, and Centre Hospitalier Universitaire (CHU) Sainte-Justine in Montréal, Canada. Perinatally HIV-1-infected children (HIV^+^), HEU children who were exposed to ART *in utero* and/or during post-natal prophylaxis and HIV uninfected unexposed control children (HIV^−^) aged 6 weeks to 19 years were enrolled between December 2008 and July 2010. No children were infected with or exposed to HIV-2 and all references in the manuscript are to HIV-1. For HIV^−^ controls, anonymous leftover blood samples from distinct children seen at BC Children’s hospital emergency department (April-June 2010) were used. Written consent was obtained from the children and/or their parents/guardians. The study was approved by the University of BC Research Ethics Board and the Children’s & Women’s Health Centre of BC Research Review Committee (H03-70356 and H04-70540) and by the Comité d’éthique de la recherche du CHU Sainte-Justine (#2872).

### Sample, Clinical and Demographic Data Collection

Venous blood was collected and shipped at room temperature to a single laboratory in Vancouver where it was stored at −80°C within 48 hours. Whole blood LTL measurements were stable up to 4 days at room temperature (data not shown). Leftover HIV^−^ control blood samples were also frozen within 48 hours of blood draw.

Except for the HIV^−^ controls, for whom only birth date and gender were available, baseline information included the children’s demographics, as well as the age of their biological parents, although paternal age was missing for approximately one quarter of subjects. Children’s ethnicity was as reported by the parent. Maternal ART history in pregnancy, as well the perinatal and postnatal ART history of HEU and HIV^+^ children were recorded. For HIV^+^ children, %CD4 nadir, %CD4 count and HIV plasma viral load (pVL) at or near the time of sample collection were collected.

### Relative Average Leukocyte Telomere Length (LTL) Assay

Total genomic DNA was extracted from 0.1 ml of whole blood using QIAamp® DNA Mini Kit (Qiagen). The relative average LTL was determined by qPCR as described [28], [29] with the following modifications. The single copy nuclear gene coding for the accessory subunit of polymerase gamma (ASPG or POLG2) was used for nuclear DNA (S) copy number determination with primers ASPG3F: 5′GAGCTGTTGACGGAAAGGAG3′ and ASPG4R: 5′CAGAAGAGAATCCCGGCTAAG3′ at 1µM. The final telomere primer concentrations were 0.3 µM for tel1b (5′CGGTTTGTTTGGGTTTGGGTTTGGGTTTGGGTTTGGGTT3′) and 0.9 µM for tel2b (5′GGCTTGCCTTACCCTTACCCTTACCCTTACCCTTACCCT3′). For both telomere (T) and (S) PCRs, 8 µL of LightCycler® 480 SYBR Green (ready-to-use hot-start PCR kit with MgCl_2_ (Roche)) master mix and 2 µL of DNA extract were added to each well. Samples were randomized and assayed in duplicate. The PCR conditions were 95°C/10 min followed by for (S) PCR, 45 cycles of 95°C/5 s, 60°C/10 s, 72°C/5 s and for (T), 45 cycles of 95°C/5 s, 54°C/30 s,72°C/1 min, in a LightCycler® 480 (Roche). The ramping temperature rate to the annealing step was set at 2.2°C/s for (S) and 1.0°C/s for (T).

Standard curves were included in each run and prepared by serial dilutions (1∶2) of pooled human blood genomic DNA, ranging from 30,000 to 469 copies of (S) and 90 to 1.4 copies of (T) and DNA concentrations ranging from ∼13.8 ng/µL to 0.22 ng/µL. LightCycler® 480 Software 1.5.0 (Roche) was used to generate the standard curve based on the maximum secondary derivative of each reaction and to determine the T and S copy numbers in each test sample. LTL was expressed as the relative T/S ratio. The intra- and inter-assay coefficients of variation were 5% and 10% respectively. We previously showed a high correlation (n = 26, r = 0.91, p<0.0001) between relative LTL measured by qPCR and lymphocyte telomere length measured by flow-fluorescence in situ hybridization [30].

### Statistical Analyses

Chi-square, Student’s t, Wilcoxon rank sum, or Kruskal-Wallis tests were used to compare the study groups’ demographic and clinical characteristics. Univariate linear regression models were used to examine the relationships of various explanatory variables with LTL. Potential age/group and age/detectable HIV pVL interactions were also explored. In addition to HIV status group, variables which were important in univariate analysis (*p*<0.15) were included in multivariate models, which were then reduced to models including only variables with *p*<0.10 in the multivariate model. Three separate models were developed: the first one for all subjects, which only considered group, age and gender; a second for HIV^+^ and HEU children, for whom more extensive demographic data were available; and a third one for HIV^+^ children only, which also included several HIV-specific parameters. Explanatory variables explored included age, gender, ethnicity, site, and parents’ ages at the time of the child’s birth. In addition to these, variables explored for the third model (HIV^+^ only) also included having a detectable HIV pVL at the time of study, highest HIV pVL ever, %CD4 count, %CD4 nadir, having had an AIDS-defining illness, length of treatment with ART, percentage of lifetime on ART, and number of ≥1 week ART interruptions. Rate of telomere attrition over time within each study group was estimated using linear regression.

To verify the validity of our method, models were also developed using the Akaike Information Criterion with finite sample size correction (AICc), the Predicted Residual Sum of Squares Statistic (PRESS), and the Schwartz Bayesian Information Criterion (SBC) (Text S1). A sensitivity analysis was conducted, omitting siblings from the data set, since their results are more likely to be correlated and violate the assumption of independence of the statistical model. One child per family was randomly selected from each of 25 sibling groups. Since paternal age was unavailable for a large number of subjects, an additional sensitivity analysis was performed to compare the final multivariate model with one including paternal age for those subjects for whom it was known. Analyses were conducted using SAS Version 9.2 (SAS).

## Results

### Study Populations

The study included 94 HIV^+^ children, 177 HEU children exposed to ART perinatally and 5 HIV^−^ children prospectively enrolled in the CARMA cohort. Anonymous blood leftover from routine blood collection was obtained from 99 HIV^−^ children. The demographic information is presented in Table 1. While HIV^−^ controls were well distributed among all ages, HIV^+^ children were older than HEU children (median age 13.3 *vs*. 1.7 years). The majority of children in both the HIV^+^ and HEU groups, according to parental identified ethnicity, were Black/African Canadians, followed by White and Aboriginal/First Nation/Metis/Inuit (referred to as Aboriginal hereafter). Of note, 158/173 Black/African and 0/22 Aboriginal children were from Montreal. Fifty-seven children had siblings, forming 25 sibling units within the study, within and across the HIV^+^ and HEU groups.

**Table 1:** Demographic characteristics of the study populations.

	HIV^+^	HEU	HIV^−^	P value^a^
	N = 94	N = 177	N = 104	
**Site, N (Vancouver/Montreal)**	40/54	37/140	104/0	<0.01
**Male gender, N (%)**	57 (61)	93 (53)	50 (48)	0.20
**Age (years) median [IQR] (range)**	13.3 [9.9–15.8] (1.1–19.0)	1.7 [0.6–4.0] (0.1–15.2)	10.6 [5.3–14.2] (0.2–19.0)	<0.01
**Ethnicity** b **, N (%)**				0.07
White	15 (16)	27 (15)	N/A	
Black/African Canadian	58 (62)	115 (65)	N/A	
Aboriginal/First Nation/Metis/Inuit	13 (14)	9 (5)	N/A	
Other	4 (4)	18 (10)	N/A	
Unknown	4 (4)	8 (5)	N/A	
**Born in Canada, N (%)**	59 (63)	177 (100)	N/A	<0.001
**Mother’s age at child’s birth (years)** c	29 [25]–[33] (17–43)	31 [28]–[36] (18–45)	N/A	<0.01
**Father’s age at child’s birth (years)** c	35 [30]–[39] (22–57)	36 [30]–[42] (19–64)	N/A	0.14

HEU, HIV-1 exposed uninfected; N/A, Not available;

a Between-group comparison by Chi-square, t or Wilcoxon rank sum test, as appropriate.

b Self-reported ethnicity; if one parent reported a non-white ethnicity, that ethnicity was assigned to the child. Ethnicity is not reported for the HIV^−^ group as no data were available for 99 of them. All Aboriginal/First nation/Metis/Inuit and 15/173 Black/African Canadian children were from the Vancouver site.

c Maternal and paternal age data were known/available for 84/94 and 67/94 of HIV^+^ children respectively. Maternal and paternal age data were known/available for 177/177 and 136/177 of HEU children respectively.

### HIV-1 and Antiretroviral Drug Exposure

The regimens used in pregnancy and their duration are described in Table 2. All 177 HEU children were exposed to ART *in utero* and/or during prophylaxis, for a median 26 and 6 weeks respectively, which closely reflects the length of treatment according to guidelines [2], [3]. Although AZT+3TC formed the backbone of ∼70% of ART regimens used in pregnancy, several other ART combinations were also used (Table 2). HIV^+^ children spent a median 55% of their lifetime on ART, and while 22% were off ART at study visit, 38% had a detectable pVL. In addition, 41% of subjects in this group never experienced an ART interruption lasting a week or longer, while others experienced up to four such breaks in treatment.

**Table 2:** ART exposure and clinical characteristics of the HIV^+^ and HEU subjects.

	HIV^+^ N = 94	HEU N = 177
**Exposed to ART, N (%)**		
*In utero*	9 (10)	176 (99)
Post-natal prophylaxis	14 (15)	177 (100)
In childhood	88 (94)	0 (0)
**Duration of ART exposure (weeks)**		
*In utero* a	0 [0–0] (0–19)	26 [16]–[38] (0–42)^b^
Post-natal prophylaxis	0 [0–0] (0–12)	6 [6–6] (2–8)
In childhood^c^	338 [174–527] (0–799)	n/a
**Type of ART exposure, N (%)**		
***In utero*** d		
Intra-partum IV AZT only	2 (22)	2 (1)
AZT mono-therapy	4 (44)	3 (2)
AZT +3TC + PI	2 (22)	114 (64)
AZT +3TC + NVP	0 (0)	9 (5)
ABC +3TC + PI	0 (0)	15 (9)
ABC +3TC + NVP	0 (0)	4 (2)
TDF + (FTC or 3TC) + PI	0 (0)	10 (6)
(D4T or ddI) +3TC + PI	0 (0)	3 (2)
Other 3 drug regimens	0 (0)	6 (3)^e^
≥4 drugs regimen	0 (0)	8 (5)^f^
Unknown regimen	1 (11)	0 (0)
***Post-natal prophylaxis (± single dose NVP)***		
AZT	9 (64)	43 (24)
AZT +3TC	2 (14)	79 (45)
AZT +3TC + NFV	3 (21)	54 (31)
ABC +3TC + NFV	0 (0)	1 (<1)
**Percentage of lifetime on ART**	55 [32–80] (0–100)	5.6 [2.8–19.6](0.8–100)^g^
**On ART at study visit, N (%)**	73 (78)	1 (<1)
**Number of subjects with lifetime ART interruptions (0/1/2/3/4) lasting >1 week**	39/27/15/5/8	0/177/0/0/0
**Detectable pVL, N (%)**	35 (37)	n/a
**If detectable, log HIV pVL at study visit (copies/mL)**	3.4 [3.0–4.3] (1.6–5.3)	n/a
**Highest log HIV pVL ever (copies/mL)**	5.0 [4.5–5.6] (1.7–7.4)	n/a
**% CD4^+^ count at study visit**	30 [25]–[37] (5–53)	N/A
**% CD4^+^ count nadir**	20 [11]–[28] (1–43)	N/A

Results are expressed as median [IQR] (range) unless otherwise indicated. N/A, Not available; n/a, not applicable; IV, intravenous; AZT, zidovudine; 3TC, lamivudine; PI, protease inhibitor; NVP, nevirapine; TDF, tenofovir, FTC, entrabicine, d4T, stavudine, ddI, didanosine, NFV, nelfinavir; T20, fuzeon.

a Refers to exposure during pregnancy, labour and delivery.

b Duration of exposure missing for 2 HEU.

c Not including post-natal prophylaxis.

d Refers to longest regimen during pregnancy (± intra-partum IV AZT, single dose NVP), 32 women underwent a regimen change during their pregnancy.

e These consisted of: 3TC+PI+NVP (N = 1); AZT+3TC+EFV (N = 1); ddI+NVP+NFV (N = 1); 3 PI (N = 1); AZT+ABC+3TC (N = 2).

f These consisted of: AZT+3TC+ABC+PI (N = 3); AZT+3TC+TDF+PI (N = 1); AZT+3TC+ddI+PI (N = 1); TDF+FTC+NVP+PI (N = 1); TDF+3TC+T20+PI (N = 1); TDF+3TC+ABC+PI (N = 1).

g Including post-natal prophylaxis.

### Relative Average Peripheral Blood Leukocyte Telomere Length (LTL)

**Figure 1:**
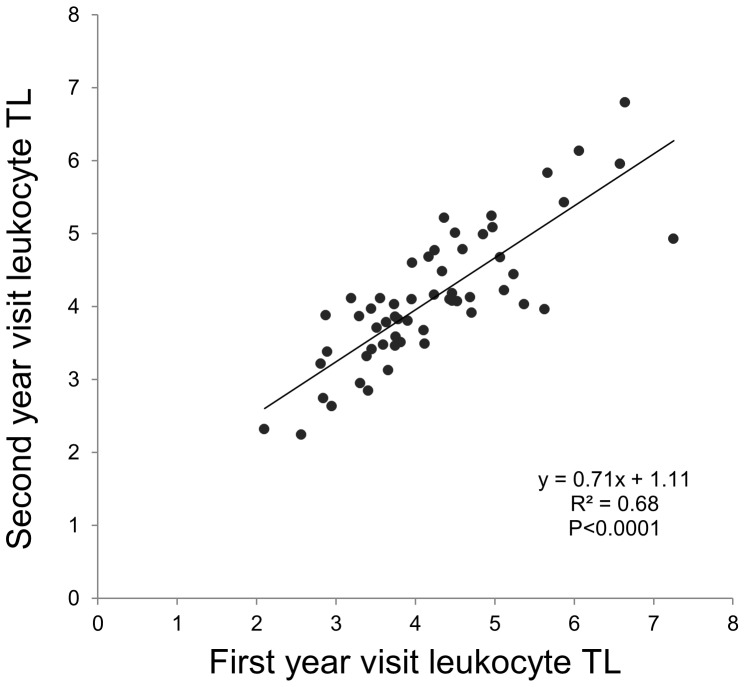
Pearson’s correlation between the leukocyte telomere length (LTL) measured in two samples collected from the same study participant one year apart.

LTL was measured by qPCR and, for a subset of children ≥8 years old, two samples collected a year apart were available and assayed. The LTL of the first visit sample was highly correlated with that measured a year later (n = 57, R^2^ = 0.68, *p*<0.0001) illustrating the longitudinal stability of the measurement (Figure 1). LTL at the second visit (average ± SD, 4.11±0.92) was approximately 2.5% shorter but not significantly different from the first visit (4.21±1.06, *p* = 0.2).

**Figure 2:**
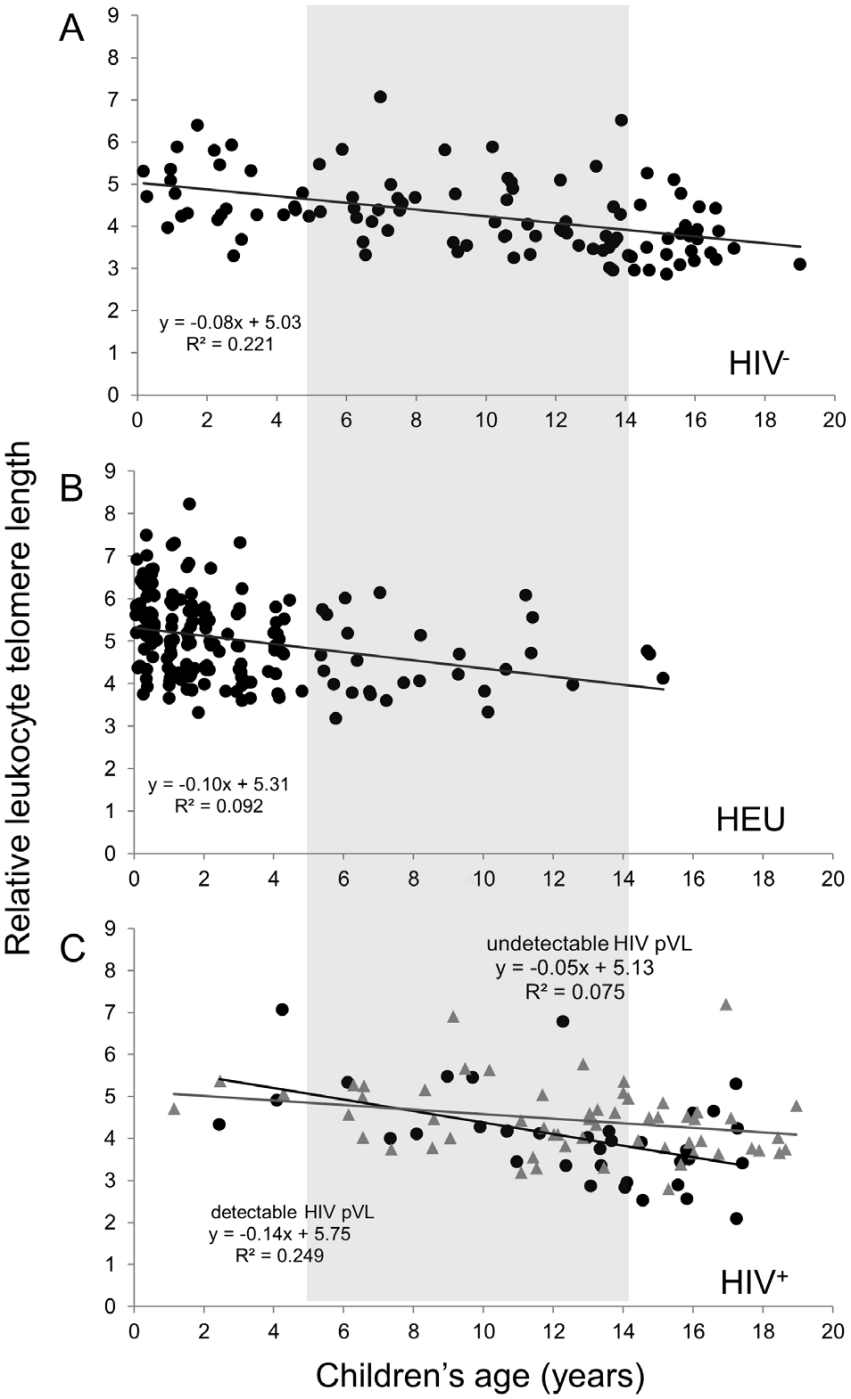
Relative leukocyte telomere length (LTL) as a function of age for the HIV^−^ (n = 104) (A), HEU (n = 177) (B) and HIV^+^ (n = 93 with known HIV plasma viral load (pVL) (C) children and youth. The light grey box identifies the 5–14 years subgroups. For the HIV^+^ group (C), subjects with an undetectable HIV pVL are depicted by grey triangles while those with a detectable HIV pVL are black circles. The equations corresponding to the linear regressions are shown on the graphs. The equation for the entire HIV^+^ group is y = −0.084×+5.335, R^2^ = 0.128.

The LTL values for the three groups as a function of age are depicted in Figure 2. A regular decline was observed during the first two decades of life of the HIV^−^ controls (Figure 2A), for a ∼33% decrease in LTL by age 19. The three groups showed similar LTL values at given ages, and similar linear regression slopes. However, the small number of young HIV^+^ and older HEU in this study limits the accurate determination of the rate of LTL decline and the comparison between these two groups over the broad age range. The regression lines in Figure 2C suggest that there may be a faster rate of LTL attrition among the HIV^+^ children who exhibited a detectable pVL at study visit compared to those with undetectable pVL (p = 0.08 for the difference between the two slopes).

A number of explanatory variables were examined for their possible association with LTL. Results of the three linear regression models are presented in Table 3. In the first multivariate model for all children (HIV^+^, HEU, and HIV^−^), older age and male gender were significantly associated with shorter LTL but HIV status group was not. Although no statistically significant interaction was seen between group and age, the group comparison was limited by the imbalance of the subjects’ ages by group. However, similar results were obtained when repeating the analysis on children aged 5–14 only (Tables S1, S2, and S3). Site (Vancouver *vs.* Montreal) was investigated as a possible explanatory variable and univariate analyses did suggest that Montreal subjects had longer LTL, something that was likely related to other differences between the sites, such as ethnicity.

**Table 3:** Linear regression models of co-variables investigated for possible association with leukocyte telomere length (LTL).

	HIV^−/^HEU/HIV^+^				HEU/HIV^+^				HIV^+^ only			
	Univariate		Multivariate R^2^ = 0.27		Univariate		Multivariate^c^ R^2^ = 0.27		Univariate		Multivariate R^2^ = 0.35	
	N = 375		N = 375		N = 271a,b		N = 237		N = 94a,b		N = 85	
	ß	P value	ß	P value	ß	P value	ß	P value	ß	P value	ß	P value
Group												
HEU *vs.* HIV^−^	0.78	<0.001	0.03	0.87	–	–	–	–	–	–	–	–
HIV^+^ *vs.* HIV^−^	0.03	0.81	0.16	0.27	–	–	–	–	–	–	–	
HEU *vs.* HIV^+^	–	–	–	–	0.75	<0.001	−0.18	0.37	–	–	–	–
Age (per year)	−0.09	<0.001	−0.09	<0.001	−0.08	<0.001	−0.08	<0.0001	−0.08	<0.001	−0.07	<0.01
Gender (Female *vs*. Male)	0.26	0.01	0.19	0.04	0.41	0.001	0.27	0.02	0.73	<0.001	0.70	<0.001
Site (Montreal *vs.* Vancouver)	0.50	<0.001	0.23	0.06	0.34	0.01	–	–	0.51	<0.01	–	–
Ethnicity^a^								0.0004		0.04		
Black *vs.* White		n.a.		n.a.	0.29	0.08	0.19	0.21	0.33	0.24	0.02	0.94
Aboriginal *vs.* White		n.a.		n.a.	−0.72	<0.01	−0.60	<0.01	−0.41	0.26	−0.58	0.07
Maternal age^b^		n.a.		n.a.	0.04	<0.01	–	–	0.003	0.89	–	–
Paternal ageb, c		n.a.		n.a.	0.03	0.001	–	–	0.03	0.13	–	–
Not on ART at visit									−0.40	0.09	–	–
Detectable pVL									−0.39	0.06	−0.51	0.007^d^
HIV pVL		n.a.		n.a.		N/A		N/A	−0.09	0.34	–	–
Number of lifetime ART interruptions >1 week		n.a.		n.a.		N/A		N/A			–	–
0 *vs*. 3/4									0.64	0.04	–	–
1 *vs.* 3/4									0.71	0.03	–	–
2 *vs.* 3/4									0.20	0.58	–	–
Percentage of lifetime on ART		n.a.		n.a.		N/A		N/A	0.002	0.56	–	–
% CD4 count		n.a.		n.a.		N/A		N/A	0.03	0.02	–	–
% CD4 nadir		n.a.		n.a.		N/A		N/A	0.008	0.37	–	–
AIDS-defining illness ever		n.a.		n.a.		N/A		N/A	0.07	0.73	–	–

A positive ß value indicates an association with longer LTL.

N/A, Not available; n/a, not applicable.

a Subjects with ethnicity Aboriginal, Black or Caucasian, N = 237/271 for HIV^+^/HEU, and N = 86/94 for HIV^+^.

b Maternal and paternal age at birth were known for N = 258/271 and N = 202/271 for HIV^+^/HEU and N = 83/94 and N = 66/94 for HIV^+^, respectively.

c In a similar multivariate model where paternal age was included (data not shown), younger paternal age showed some association with shorter LTL (p = 0.06). The coefficients and p-values for the remaining explanatory variables were very similar between these two models.

d Since not on ART at visit and having a detectable pVL at visit are correlated, the variable with the lowest p value univariately was chosen for inclusion into the multivariable model. Models developed using AICc, PRESS, and SBC showed very similar results (supporting information).

In the second model, which included HEU and HIV^+^ children only, additional explanatory variables investigated included maternal age, paternal age, and ethnicity. In univariate analyses, HIV^+^ status (*vs.* HEU), older age, male gender, Vancouver site, Aboriginal ethnicity, younger maternal and younger paternal age were all associated with shorter LTL (p<0.05). However, in the final multivariate model that excluded parental ages because of multiple missing values, HIV status (HIV^+^ vs. HEU) was not associated with shorter LTL but older child age, male gender and Aboriginal ethnicity remained associated with shorter LTL (Table 3). Similarly, when analysis was restricted to children aged 5–14 years of age in whom the mean ages of the groups were less than 2 years apart, there was no association between LTL and HIV status (Table S3).

In the third model which included HIV^+^ children only, older age, male gender, Vancouver site, and lower %CD4 cell count were univariately associated with shorter LTL (p<0.05). Having a detectable pVL and a greater number of ART interruptions lasting >1 week were weakly associated with shorter LTL based on p-values (Table 3). In the multivariate model, only older age, male gender and a detectable pVL remained significantly associated with shorter LTL, with Aboriginal ethnicity still showing a weak relationship (p = 0.07). A significant (p = 0.02), age/detectable pVL interaction was detected, suggesting a faster rate of decline in LTL for HIV^+^ subjects if they had a detectable pVL (Figure 2C). However, due to the sparseness of the data, this model was viewed as an exploratory analysis, and the interaction term was not included in the final model.

**Figure 3:**
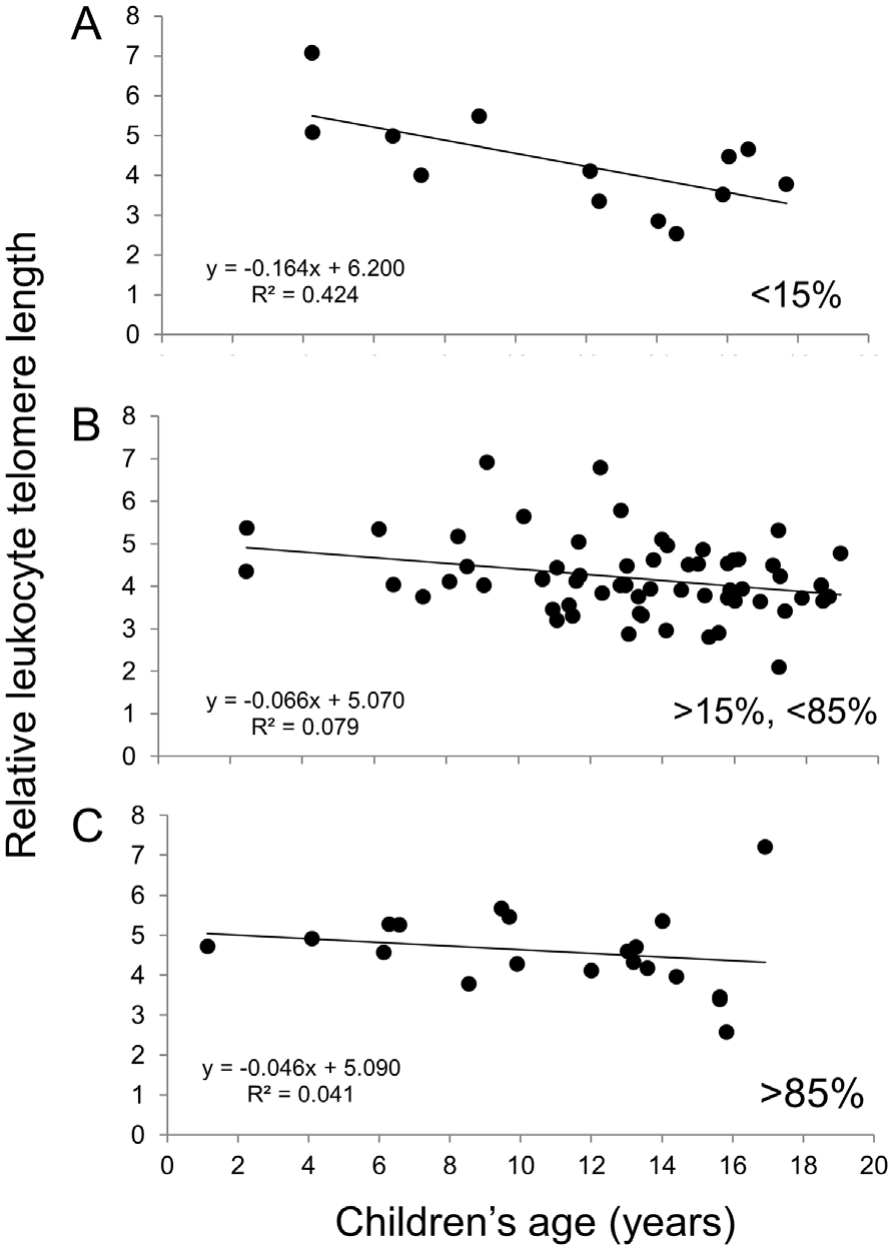
Relative leukocyte telomere length (LTL) as a function of age for the HIV^+^ children according to the percentage of their life spent on ART: less than 15% (A), between 15 and 85% (B) and greater than 85% (C). The equations corresponding to the linear regressions are shown on the graphs.

**Figure 4:**
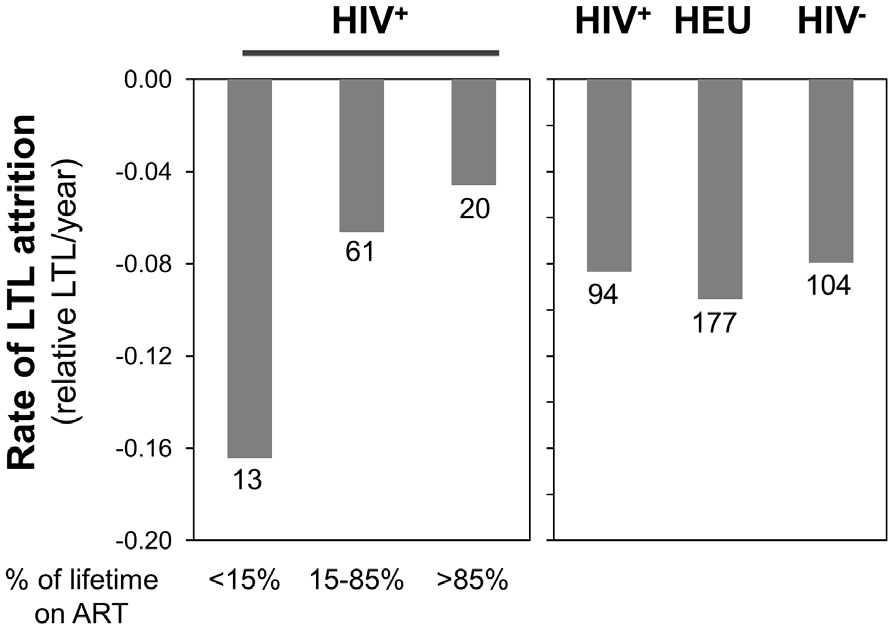
Summary of the rates of telomere attrition determined by the slope of the linear regressions for all children in the HIV^+^, HEU and HIV^−^ groups (right panel), as well as for the HIV^+^ group dichotomized according to the percentage of the children’s life spent on ART: less than 15%, between 15 and 85%, and greater than 85% (left panel). The number of individual represented in each bar is indicated below it.

Finally, Figure 2 indicates that the rates of telomere decline are similar between the three groups, in agreement with the statistical model. Figures 3 and 4 suggest that children who received ART for less than 15% of their life (6/13 ART-naive) show a rate of telomere attrition almost twice as fast as that of the HIV^+^ group as a whole and three times faster than children who received ART for more than 85% of their life. Nevertheless, in the third multivariate model (for HIV^+^ children only), the percentage of lifetime on ART, as a continuous variable, was not independently associated with LTL (Tables 3 and S3).

Because 57 children had siblings within the study, distributed within and across HEU and HIV^+^ groups, a sensitivity analysis was performed, including only one randomly selected child from each of the 25 families. It showed that most of the model selection results were the same as in the primary analysis as were those developed using AICc, PRESS, and SBC statistics to guide model selection.

## Discussion

We measured LTL to investigate aging phenomena at the cellular level in a cohort of perinatally infected HIV^+^ children, HEU children exposed to NRTIs either *in utero* and/or early in life, and HIV^−^ control children.

The group rates of LTL attrition were similar for HIV^+^, HEU and HIV^−^ children (Figure 2) and no association between HIV status and LTL was seen in linear regression models. Given the imbalance in age distribution between the HEU and HIV^+^ groups, we repeated the analyses with sub-groups closer in age and obtained similar results. These are certainly positive and reassuring data for HIV^+^ and HEU children. As expected, advancing age showed the strongest association with shorter LTL, with an overall rate of telomere attrition of 1.5–2.5% per year during the first 19 years of life. Smaller pediatric studies (n = 9 or 10) have reported a rapid loss of LTL in healthy children during the first 3–4 years of life followed by a leveling in middle-age and a second phase of decline later in life [31], [32]. In this larger study, we did not see evidence of a biphasic decline in the HIV^−^ control children. Reflecting successful prevention of mother-to-child HIV transmission and the fact that HEUs are inconsistently followed beyond age 2, HIV^+^ children were generally older than HEU ones.

An association between male gender and shorter LTL was observed (Tables 3 and S3). The fact that female gender protects telomeres has been reported in numerous adult studies [33], [34] and recently in adolescents [35] but no such gender-based difference was previously seen in newborns [36]. A protective effect of estrogen on telomerase expression and activity in women, as well as increased inflammation and oxidative stress in males have been suggested as possible biological explanations for this gender-related difference [37]. To our knowledge, our study is the first to report this association in children. It is noteworthy that the gender effect on LTL was more pronounced in older children, as seen in the third (HIV^+^ only) model (Table 3). However, gender remained an explanatory variable in the first multivariate model, in which 60% of subjects were under 9 years of age, hence mostly pre-pubertal, suggesting that factors other than sex hormones may play a role here.

Subjects of Aboriginal ethnicity had (HEU/HIV^+^ model) or tended toward (HIV^+^ only model) shorter LTL than white subjects. This relationship between ethnicity and LTL may be confounded by uneven distribution of ethnicity in the HEU/HIV^+^ models. However, the fact that the effect persists in the HIV^+^ models may suggest the involvement of environmental and/or genetic factors, something this study was neither designed nor powered to investigate. Site was associated with LTL in univariate models, with subjects from Montreal having longer LTL than those from Vancouver, but this did not persist in multivariate models, suggesting it acted as a univariate surrogate for other variables such as ethnicity.

Numerous studies have established that offspring LTL is more strongly associated with paternal than maternal age at birth, whereby the progeny of older fathers have longer LTL [38], [39], [40]. Our results also suggested such association although the variable did not emerge in multivariate models. Biological father’s age was challenging to collect in this cohort and data were missing for almost a quarter of subjects. However, given that the results from the sensitivity analysis comparing the final HEU/HIV^+^ model with one which included this variable were very similar, it appears that in the presence of other information, paternal age was not one of the most important predictors.

A key finding was that among HIV^+^ children, those with detectable plasma HIV-1 RNA had shorter LTL compared to their peers. Similarly, among the 5–14 year old children, not being on ART was more strongly associated with shorter LTL. This strongly suggests that uncontrolled HIV viremia rather than exposure to ART may be responsible for accelerated telomere attrition in HIV^+^ individuals. In an attempt to further explore how circulating virus may lead to shorter LTL, percentage of lifetime on ART was included in the models as a potential surrogate for the amount of HIV-related inflammation the subject might have been exposed to. No statistically significant relationship emerged from the regression analyses but the rates of telomere attrition appeared higher in HIV^+^ children who received lesser amounts of ART. This is consistent with the recent observation that telomere length in naive CD4^+^ T cells of young ART-naive individuals approaches that seen decades later in HIV^−^ individuals, something that is partially reconstituted by ART [41]. Our results are also consistent with chronic immune activation leading to activation of B and T lymphocytes [42], as this systemic immune activation is decreased in HIV^+^ individuals on effective ART [43], [44].

In addition, as intermittent ART has been associated with higher mortality [45] and higher levels of inflammatory markers [46], the number of ART interruptions was explored. Although having fewer ART interruptions was univariately associated with longer LTL, this association did not persist once age and other variables were considered. Further studies are needed to confirm the link between pVL and LTL, to determine whether short-term or long-term uncontrolled viremia best explains this association, to define whether the LTL decline is transient or permanent, and to sort out whether it is related to inflammation and oxidative stress [11], [44], [47] and/or immune cell proliferation [48], [49].

Among the strengths of the present study are its sample size- it is the largest study of LTL in perinatally HIV infected children and youth to date- and the fact that duration of HIV-1 infection was well defined. In addition, lifestyle or environmental factors such as smoking, drugs or alcohol use and other health-modulating habits that can affect LTL are less likely to have exerted their influence. Finally, HEU and HIV^+^ subjects in this study likely had similar familial and socio-economic environments, further diminishing the potential influence of these factors.

A limitation of this study is the unequal distribution of age between the groups, something that a larger study may address. However, the analysis of sub-groups comprising only children aged 5 to 14 years showed the same lack of difference by HIV-1 status.

In conclusion, LTL in HIV^+^ and HEU children follows a generally similar pattern to that in the HIV^−^ control group, a reassuring observation. Older children and those of male gender had shorter LTL. Among HIV^+^ subjects, having a detectable HIV pVL or not being on ART was strongly associated with shorter LTL. It will be of interest to further characterize the frequencies and telomere lengths of immune cell subtypes within these pediatric populations, including CD8^+^CD28^−^ T cells [42], [50]. Future studies, longitudinal or with larger sample size, will also be required to fully characterize the relationship between HIV-1 viremia and telomere dynamics and determine whether similar associations can also be observed in adults.

## Supporting Information

Table S1 Demographic characteristics of the study populations aged 5–14 years.
(DOCX)

Table S2 ART exposure and clinical characteristics of the HIV^+^ and HEU subjects aged 5–14.
(DOCX)

Table S3 Linear regression models of co-variables investigated for possible association with leukocyte telomere length (LTL) among the subgroup of children aged 5–14 years. A positive ß value indicates an association with longer LTL.
(DOCX)

Text S1 Additional statistical analyses performed in addition to those presented herein.
(DOCX)

## References

[1] Gray GE, McIntyre JA (2007). HIV and pregnancy.. Bmj.

[2] World Health Organization (2010). Antiretroviral drugs for treating pregnant women and preventing HIV infection in infants.. Geneva, Switzerland: World Health Organization.

[3] AIDSinfo website (2011). Recommendations for Use of Antiretroviral Drugs in Pregnant HIV-1-Infected Women for Maternal Health and Interventions to Reduce Perinatal HIV Transmission in the United States.. http://aidsinfo.nih.gov/contentfiles/PerinatalGL.pdf.

[4] Forbes JC, Alimenti AM, Singer J, Brophy JC, Bitnun A (2012). A national review of vertical HIV transmission.. AIDS.

[5] World Health Organization website.. http://www.who.int/hiv/topics/paediatric/en/index.html.

[6] Chappuy H, Treluyer JM, Jullien V, Dimet J, Rey E (2004). Maternal-fetal transfer and amniotic fluid accumulation of nucleoside analogue reverse transcriptase inhibitors in human immunodeficiency virus-infected pregnant women.. Antimicrob Agents Chemother.

[7] Zhu H, Belcher M, van der Harst P (2011). Healthy aging and disease: role for telomere biology?. Clin Sci (Lond).

[8] Majerska J, Sykorova E, Fajkus J (2011). Non-telomeric activities of telomerase.. Mol Biosyst.

[9] Wright WE, Piatyszek MA, Rainey WE, Byrd W, Shay JW (1996). Telomerase activity in human germline and embryonic tissues and cells.. Dev Genet.

[10] Collins K, Mitchell JR (2002). Telomerase in the human organism.. Oncogene.

[11] Effros RB (2011). Telomere/telomerase dynamics within the human immune system: effect of chronic infection and stress.. Exp Gerontol.

[12] Bestilny LJ, Gill MJ, Mody CH, Riabowol KT (2000). Accelerated replicative senescence of the peripheral immune system induced by HIV infection.. Aids.

[13] Deeks SG (2011). HIV infection, inflammation, immunosenescence, and aging.. Annu Rev Med.

[14] Gillis AJ, Schuller AP, Skordalakes E (2008). Structure of the Tribolium castaneum telomerase catalytic subunit TERT.. Nature.

[15] Peng Y, Mian IS, Lue NF (2001). Analysis of telomerase processivity: mechanistic similarity to HIV-1 reverse transcriptase and role in telomere maintenance.. Mol Cell.

[16] Yamaguchi T, Takayama Y, Saito M, Ishikawa F, Saneyoshi M (2001). Telomerase-inhibitory effects of the triphosphate derivatives of some biologically active nucleosides.. Nucleic Acids Res.

[17] Hukazelie KC, Wong J (2011). In vitro and in vivo inhibition studies of human telomerase by HIV reverse transcriptase. 20th Annual Canadian Conference on HIV/AIDS Research.. Toronto, Ontario, Canada: Can J Infect Dis Med Microbiol.

[18] Strahl C, Blackburn EH (1996). Effects of reverse transcriptase inhibitors on telomere length and telomerase activity in two immortalized human cell lines.. Mol Cell Biol.

[19] Brown T, Sigurdson E, Rogatko A, Broccoli D (2003). Telomerase inhibition using azidothymidine in the HT-29 colon cancer cell line.. Ann Surg Oncol.

[20] Liu X, Takahashi H, Harada Y, Ogawara T, Ogimura Y (2007). 3'-Azido-2',3'-dideoxynucleoside 5'-triphosphates inhibit telomerase activity in vitro, and the corresponding nucleosides cause telomere shortening in human HL60 cells.. Nucleic Acids Res.

[21] Strahl C, Blackburn EH (1994). The effects of nucleoside analogs on telomerase and telomeres in Tetrahymena.. Nucleic Acids Res.

[22] Olivero OA, Fernandez JJ, Antiochos BB, Wagner JL, St Claire ME (2002). Transplacental genotoxicity of combined antiretroviral nucleoside analogue therapy in Erythrocebus patas monkeys.. J Acquir Immune Defic Syndr.

[23] Bazarbachi A, Plumelle Y, Carlos Ramos J, Tortevoye P, Otrock Z (2010). Meta-analysis on the use of zidovudine and interferon-alfa in adult T-cell leukemia/lymphoma showing improved survival in the leukemic subtypes.. J Clin Oncol.

[24] Datta A, Bellon M, Sinha-Datta U, Bazarbachi A, Lepelletier Y (2006). Persistent inhibition of telomerase reprograms adult T-cell leukemia to p53-dependent senescence.. Blood.

[25] De Meyer T, Rietzschel ER, De Buyzere ML, Van Criekinge W, Bekaert S (2011). Telomere length and cardiovascular aging: the means to the ends?. Ageing Res Rev.

[26] Sanders JL, Fitzpatrick AL, Boudreau RM, Arnold AM, Aviv A (2011). Leukocyte Telomere Length Is Associated With Noninvasively Measured Age-Related Disease: The Cardiovascular Health Study.. J Gerontol A Biol Sci Med Sci.

[27] Willeit P, Willeit J, Mayr A, Weger S, Oberhollenzer F (2010). Telomere length and risk of incident cancer and cancer mortality.. JAMA.

[28] Cawthon RM (2002). Telomere measurement by quantitative PCR.. Nucleic Acids Res.

[29] Gil ME, Coetzer TL (2004). Real-time quantitative PCR of telomere length.. Mol Biotechnol.

[30] Oliveira LSB, Hukezalie K, Maan E, Gadawski I, Vulto I (2010). Telomere length measurement in fresh and frozen cord blood and placenta by quantitative PCR: comparison with other methods.. Can J Inf Dis Med Microbiol.

[31] Frenck RW, Blackburn EH, Shannon KM (1998). The rate of telomere sequence loss in human leukocytes varies with age.. Proc Natl Acad Sci U S A.

[32] Zeichner SL, Palumbo P, Feng Y, Xiao X, Gee D (1999). Rapid telomere shortening in children.. Blood.

[33] Mayer S, Bruderlein S, Perner S, Waibel I, Holdenried A (2006). Sex-specific telomere length profiles and age-dependent erosion dynamics of individual chromosome arms in humans.. Cytogenet Genome Res.

[34] Moller P, Mayer S, Mattfeldt T, Muller K, Wiegand P (2009). Sex-related differences in length and erosion dynamics of human telomeres favor females.. Aging (Albany NY).

[35] Zhu H, Wang X, Gutin B, Davis CL, Keeton D (2011). Leukocyte telomere length in healthy Caucasian and African-American adolescents: relationships with race, sex, adiposity, adipokines, and physical activity.. J Pediatr.

[36] Okuda K, Bardeguez A, Gardner JP, Rodriguez P, Ganesh V (2002). Telomere length in the newborn.. Pediatr Res.

[37] Aviv A (2002). Telomeres, sex, reactive oxygen species, and human cardiovascular aging.. J Mol Med.

[38] De Meyer T, Rietzschel ER, De Buyzere ML, De Bacquer D, Van Criekinge W (2007). Paternal age at birth is an important determinant of offspring telomere length.. Hum Mol Genet.

[39] Kimura M, Cherkas LF, Kato BS, Demissie S, Hjelmborg JB (2008). Offspring's leukocyte telomere length, paternal age, and telomere elongation in sperm.. PLoS Genet.

[40] Nordfjall K, Svenson U, Norrback KF, Adolfsson R, Roos G (2010). Large-scale parent-child comparison confirms a strong paternal influence on telomere length.. Eur J Hum Genet.

[41] Rickabaugh TM, Kilpatrick RD, Hultin LE, Hultin PM, Hausner MA (2011). The dual impact of HIV-1 infection and aging on naive CD4 T-cells: additive and distinct patterns of impairment.. PLoS One.

[42] Lin J, Epel E, Cheon J, Kroenke C, Sinclair E (2010). Analyses and comparisons of telomerase activity and telomere length in human T and B cells: insights for epidemiology of telomere maintenance.. J Immunol Methods.

[43] Brenchley JM, Price DA, Schacker TW, Asher TE, Silvestri G (2006). Microbial translocation is a cause of systemic immune activation in chronic HIV infection.. Nat Med.

[44] Hunt PW (2012). HIV and Inflammation: Mechanisms and Consequences.. Curr HIV/AIDS Rep.

[45] El-Sadr WM, Grund B, Neuhaus J, Babiker A, Cohen CJ (2008). Risk for opportunistic disease and death after reinitiating continuous antiretroviral therapy in patients with HIV previously receiving episodic therapy: a randomized trial.. Ann Intern Med.

[46] Kuller LH, Tracy R, Belloso W, De Wit S, Drummond F (2008). Inflammatory and coagulation biomarkers and mortality in patients with HIV infection.. PLoS Med.

[47] Trudeau MA, Wong JM (2010). Genetic Variations in Telomere Maintenance, with Implications on Tissue Renewal Capacity and Chronic Disease Pathologies.. Curr Pharmacogenomics Person Med.

[48] van de Berg PJ, Griffiths SJ, Yong SL, Macaulay R, Bemelman FJ (2010). Cytomegalovirus infection reduces telomere length of the circulating T cell pool.. J Immunol.

[49] Pawelec G, Akbar A, Caruso C, Effros R, Grubeck-Loebenstein B (2004). Is immunosenescence infectious?. Trends Immunol.

[50] Dock JN, Effros RB (2011). Role of CD8 T Cell Replicative Senescence in Human Aging and in HIV-mediated Immunosenescence.. Aging Dis.

