# Supplementary material for: Leukocyte Telomere Length in HIV-Infected and HIV-Exposed Uninfected Children: Shorter Telomeres with Uncontrolled HIV Viremia
Source: PLoS One. 2012 Jul 16;7(7):e39266. doi: 10.1371/journal.pone.0039266 (PMC3397986; doi:10.1371/journal.pone.0039266)
Supplement: Table S1 — Demographic characteristics of the study populations aged 5–14 years. (DOCX) [file pone.0039266.s001.docx]

Table S1. Demographic characteristics of the study populations aged 5-14 years.

|  | **HIV^+^ N=50** | **HEU N=26** | **HIV^-^ N=52** | **P value** ^a^ |
| --- | --- | --- | --- | --- |
| **Site, N (Vancouver/Montreal)** | 19/31 | 3/23 | 52/0 | <0.01 |
| **Male gender, N (%)** | 29 (58) | 14 (54) | 25 (48) | 0.60 |
| **Age (years) median [IQR] (range)** | 11.5 [9.0-13.0] (6.1-14.0) | 7.1 [6.0-10.0] (5.4-12.6) | 10.6 [7.4-12.5] (5.2-13.9) | <0.01 |
| **Ethnicity** ^b^**, N (%)** |  |  |  | 0.35 |
| White | 3 (6) | 1 (4) | N/A |  |
| Black/African Canadian | 36 (72) | 18 (69) | N/A |  |
| Aboriginal/First nation/Metis/Inuit | 6 (12) | 1 (4) | N/A |  |
| Other | 4 (8) | 3 (12) | N/A |  |
| Unknown | 1 (2) | 3 (12) | N/A |  |
| **Born in Canada, N (%)** | 29 (58) | 26 (100) | N/A | <0.001 |
| **Mother’s age at child’s birth (years)** ^c^ | 29 [26-33] (17-43) | 31 [29-36] (20-40) | N/A | 0.04 |
| **Father’s age at child’s birth (years)** ^c^ | 36 [30-40] (22-57) | 39 [34-43] (20-49) | N/A | 0.127 |

HEU, HIV-exposed uninfected; N/A, Not available;

^a^ Between-group comparison by Chi-square, t-test, Wilcoxon rank sum test or Fisher’s exact test, as appropriate.

^b^ Self-reported ethnicity; if one parent reported a non-white ethnicity, that ethnicity was assigned to the child. Ethnicity is not reported for the HIV^-^ group as no data were available for most of them. All Aboriginal/First nation/Metis/Inuit and 9/54 Black/African Canadian children were from the Vancouver site.

^c^ Maternal and paternal age data were known/available for 43/50 and 36/50 of HIV^+^ children respectively. Maternal and paternal age data were known/available for 25/26 and 20/26 of HEU children respectively.
