# Supplementary material for: Leukocyte Telomere Length in HIV-Infected and HIV-Exposed Uninfected Children: Shorter Telomeres with Uncontrolled HIV Viremia
Source: PLoS One. 2012 Jul 16;7(7):e39266. doi: 10.1371/journal.pone.0039266 (PMC3397986; doi:10.1371/journal.pone.0039266)
Supplement: Table S2 — ART exposure and clinical characteristics of the HIV+ and HEU subjects aged 5–14. (DOCX) [file pone.0039266.s002.docx]

Table S2. ART exposure and clinical characteristics of the HIV^+^ and HEU subjects aged 5-14.

|  | **HIV^+^ N=50** | **HEU N=26** |
| --- | --- | --- |
| **Exposed to ART, N (%)** |  |  |
| *In utero* | 7 (14) | 26 (100) |
| Post-natal prophylaxis | 11 (22) | 26 (100) |
| In childhood | 47 (94) | 0 (0) |
| **Duration of ART exposure (weeks)** |  |  |
| *In utero* ^a^ | 0 [0-0] (0-19) | 20 [12-34] (0-42) ^b^ |
| Post-natal prophylaxis | 0 [0-0] (0-12) | 6 [6-6] (6-6) |
| In childhood ^c^ | 295 [174-432] (0-693) | n/a |
| **Percentage of lifetime on ART** | 54 [32-87] (0-100) | 1.6 [1.1-1.9](0.9-2.1) ^d^ |
| **On ART at study visit, N (%)** | 40 (80) | 1 (4) |
| **Detectable pVL, N (%)** | 17 (34) | n/a |
| **If detectable, log HIV pVL at study visit (copies/mL)** | 1.7 [1.6-3.1] (1.6-4.4) | n/a |
| **Highest log HIV pVL ever (copies/mL)** | 5.0 [4.5-5.7] (3.1-7.4) | n/a |
| **CD4^+^ count at study visit** | 692[540-880] (85-1537) | N/A |
| **CD4^+^ count nadir** | 385 [250-550] (5-1080) | N/A |

Results are expressed as median [IQR] (range) unless otherwise indicated. N/A, Not available; n/a, not applicable

^a^ Refers to exposure during pregnancy, labour and delivery

^b^ Duration of exposure missing for 2 HEU

^c^ Not including post-natal prophylaxis

^d^ Including post-natal prophylaxis
