# Supplementary material for: Leukocyte Telomere Length in HIV-Infected and HIV-Exposed Uninfected Children: Shorter Telomeres with Uncontrolled HIV Viremia
Source: PLoS One. 2012 Jul 16;7(7):e39266. doi: 10.1371/journal.pone.0039266 (PMC3397986; doi:10.1371/journal.pone.0039266)
Supplement: Table S3 — Linear regression models of co-variables investigated for possible association with leukocyte telomere length (LTL) among the subgroup of children aged 5–14 years. A positive ß value indicates an association with longer LTL. (DOCX) [file pone.0039266.s003.docx]

Table S3. Linear regression models of co-variables investigated for possible association with leukocyte telomere length (LTL) among the subgroup of children aged 5-14 years. A positive ß value indicates an association with longer LTL.

|  | HIV^-^/HEU/HIV^+^ | | | | HEU/HIV^+^ | | | | HIV^+^ only | | | |
| --- | --- | --- | --- | --- | --- | --- | --- | --- | --- | --- | --- | --- |
|  | Univariate | | Multivariate R^2^=0.09 | | Univariate | | Multivariate^c^ R^2^=0.23 | | Univariate | | Multivariate R^2^=0.35 | |
|  | N=128 | | N=128 | | N=76^a,b^ | | N=76 | | N=50 ^a,b^ | | N=50 | |
|  | ß | P value | ß | P value | ß | P value | ß | P value | ß | P value | ß | P value |
| Group |  |  |  |  |  |  |  |  |  |  |  |  |
| HEU *vs.* HIV^-^ | 0.24 | 0.25 | --- | --- | --- | --- | --- | --- | --- | --- | --- | --- |
| HIV^+^ *vs.* HIV^-^ | 0.14 | 0.41 | --- | --- | --- | --- | --- | --- | --- | --- | --- | --- |
| HEU *vs.* HIV^+^ | --- | --- | --- | --- | 0.10 | 0.64 | -0.01 | 0.98 | --- | --- | --- | --- |
| Age (per year) | -0.07 | 0.012 | -0.06 | 0.03 | -0.05 | 0.14 | -0.08 | 0.2 | -0.08 | 0.11 | -0.01 | 0.82 |
| Gender (Female *vs*. Male) | 0.23 | 0.13 | 0.23 | 0.13 | 0.38 | 0.06 | 0.14 | 0.64 | 0.54 | 0.03 | 0.74 | 0.01 |
| Site (Montreal *vs.* Vancouver) | 0.32 | 0.04 | 0.31 | 0.04 | 0.40 | 0.07 | -0.66 | 0.23 | 0.27 | 0.30 | ----- | --- |
| Ethnicity ^a^ |  |  |  |  |  |  |  |  |  | 0.19 |  |  |
| Black *vs.* Caucasian |  | n.a. |  | n.a. | 0.44 | 0.33 | 0.67 | 0.27 | 0.39 | 0.46 | 0.15 | 0.77 |
| Aboriginal *vs.* Caucasian |  | n.a. |  | n.a. | -0.42 | 0.44 | -0.34 | 0.63 | -0.29 | 0.64 | -0.29 | 0.61 |
| Maternal age ^b^ |  | n.a. |  | n.a. | 0.01 | 0.62 | 0.00 | 1.0 | -0.012 | 0.67 | --- | --- |
| Paternal age ^b^ |  | n.a. |  | n.a. | 0.03 | 0.08 | 0.94 | 0.28 | 0.02 | 0.24 | --- | --- |
| Not on ART at visit |  |  |  |  |  |  |  |  | -0.58 | 0.06 | -0.80 | 0.02 |
| Detectable pVL |  |  |  |  |  |  |  |  | -0.27 | 0.30 | ---- | -- |
| HIV pVL |  | n.a. |  | n.a. |  | N/A |  | N/A | -0.10 | 0.43 | --- | --- |
| Number of lifetime ART interruptions >1 week |  | n.a. |  | n.a. |  | N/A |  | N/A |  | 0.36 | --- | --- |
| 0 *vs*. 3/4 |  |  |  |  |  |  |  |  | -0.28 | 0.51 | --- | --- |
| 1 *vs.* 3/4 |  |  |  |  |  |  |  |  | -0.21 | 0.64 | --- | --- |
| 2 *vs.* 3/4 |  |  |  |  |  |  |  |  | -0.77 | 0.12 | --- | --- |
| Percentage of lifetime on ART |  | n.a. |  | n.a. |  | N/A |  | N/A | 0.006 | 0.16 | --- | --- |
| CD4 count |  | n.a. |  | n.a. |  | N/A |  | N/A | 0.00 | 0.99 | --- | --- |
| CD4 nadir |  | n.a. |  | n.a. |  | N/A |  | N/A | 0.001 | 0.20 | --- | --- |
| AIDS-defining illness ever |  |  |  |  |  |  |  |  | 0.43 | 0.10 | --- | --- |

N/A, Not available; n/a, not applicable

^a^ Subjects with ethnicity Aboriginal, Black or Caucasian, N=65/76 for HIV^+^/HEU, and N=45/50 for HIV^+^

^b^ Maternal and paternal age at birth were known for N=68/76 and N=56/76 for HIV^+^/HEU and N=68/76 and N=56/76 for HIV^+^, respectively.

^c^ In a similar multivariate model where paternal age was included (data not shown), younger paternal age showed some association with shorter LTL (p=0.06). The coefficients and p-values for the remaining explanatory variables were very similar between these two models.

^d^  Univariate was actually adjusted for age and an age*% lifetime on ART (categorized)
