# Supplementary material for: Leukocyte Telomere Length in HIV-Infected and HIV-Exposed Uninfected Children: Shorter Telomeres with Uncontrolled HIV Viremia
Source: PLoS One. 2012 Jul 16;7(7):e39266. doi: 10.1371/journal.pone.0039266 (PMC3397986; doi:10.1371/journal.pone.0039266)
Supplement: Text S1 — Additional statistical analyses performed in addition to those presented herein. (DOCX) [file pone.0039266.s004.docx]

**Text S1. Additional statistical analyses**

**Additional analyses on all subjects:**

A sensitivity analysis was conducted whereby models were selected using proc glmselect in SAS 9.2 with the following methods:

1. stepwise selection using 0.15 as the criterion, with the choice of final model based on PRESS (the predicted residual sum of squares)
2. backwards selection using AICC (the corrected Akaike Information Criterion), with the choice of final model based on AICC
3. stepwise selection using SBC (the Schwartz Bayesian Information Criterion)), with the choice of final model based on SBC.

The same candidate variables were considered:

Model 1) HIV^+^/HEU/HIV^-^ model: group (forced into model), age, gender, and site

Model 2) HIV^+^/HEU model: group (forced into model), age, gender, ethnicity, maternal age at birth (paternal age at birth omitted due to large number of missing values)

Model 3) HIV^+^ only model: age, gender, ethnicity, detectable viral load (yes/no), percentage of life on ART, percent CD4 nadir, AIDS-defining illness ever (yes/no)

**Results**

**HIV^+^/HEU/HIV^-^** (n=375)

1. identical choice of variables to those shown in Table 3; PRESS = 288.52
2. identical choice of variables to those shown in Table 3; AICC = 278.76
3. all variables in final model as shown in Table 3, but gender not included; R^2^ (adj) = 0.26; SBC = ‑87.25

**HIV^+^/HEU** (n=237)

1. identical choice of variables to those shown in Table 3; PRESS = 178.49
2. identical choice of variables to those shown in Table 3; AICC = 174.12
3. identical choice of variables to those shown in Table 3; SBC = ‑37.20

**HIV^+^ only** (n=85)

1. identical choice of variables to those shown in Table 3; PRESS = 60.07
2. all variables in final model as shown in Table 3, with the addition of AIDS-defining illness ever (yes/no); R^2^ (adj) = 0.34; AICC = 58.35
3. all variables in final model as shown in Table 3, but ethnicity not included; R^2^ (adj) = 0.30; SBC = ‑17.41

**Analysis on subjects aged 5-14 years of age:**

To address the unequal distribution of age between the groups, the analyses were repeated on a narrower range of ages, including children aged 5 to 14 years. Demographic characteristics are presented in Table S1 while HIV-related characteristics are shown in Table S2. Once again, three models were built with the same variables as for the ones including all subjects, except that instead of % CD4 cell count and % CD4 nadir, absolute CD4 counts and CD4 nadir were considered since the children were older. Each variable was examined univariately, and included as candidate in the multivariable model when p<0.15, except for the third model (HIV^+^ only) for which, given the smaller sample size, important variables were considered in the multivariable model if p<0.2.

Although the median ages were closer to one another, age remained statistically different between the 3 groups. Results from the 3 models are presented in Table S3.

Model 1: HIV+/HEU/HIV-

In the univariate analyses, HIV status was not associated with LTL but older age and Vancouver site were associated with shorter LTL. The same variable remained associated with LTL in the multivariate model. Once again, ethnicity may explain the site association here but gender was not.

Model 2: HIV+/HEU

With the ages more closely distributed between groups, HIV status was no longer associated with LTL univariately. In the multivariate model, none of the variables was statistically associated with LTL.

Model 3: HIV+ only

In univariate analyses, older age, male gender, ethnicity, percentage of life on ART, not being on ART at visit and not having had AIDS-defining illness produced p values below 0.2 and were considered in the multivariate model.

In the final model, only male gender and not being on ART at visit were significantly associated with shorter LTL. The main difference between this model and the third model on all subjects is that, as the ages are distributed over a narrower range, older age is no longer associated with LTL.
